# *EGenBio*: A Data Management System for Evolutionary Genomics and Biodiversity

**DOI:** 10.1186/1471-2105-7-S2-S7

**Published:** 2006-09-26

**Authors:** Laila A Nahum, Matthew T Reynolds, Zhengyuan O Wang, Jeremiah J Faith, Rahul Jonna, Zhi J Jiang, Thomas J Meyer, David D Pollock

**Affiliations:** 1 Department of Biological Sciences, Biological Computation and Visualization Center, Louisiana State University, Baton Rouge, LA 70803 USA; 2 Josephine Bay Paul Center, Marine Biological Laboratory, Woods Hole, MA 02543, USA; 3 Bioinformatics Program, Boston University, Boston, MA 02215, USA; 4 Division of Developmental Disabilities, Arizona State Department, Phoenix, AZ 85012 USA; 5 Department of Biochemistry and Molecular Genetics, University of Colorado School of Medicine, Aurora, CO 80045, USA

## Abstract

**Background:**

Evolutionary genomics requires management and filtering of large numbers of diverse genomic sequences for accurate analysis and inference on evolutionary processes of genomic and functional change. We developed *E*volutionary *Gen*omics and *Bio*diversity (*EGenBio*; ) to begin to address this.

**Description:**

*EGenBio *is a system for manipulation and filtering of large numbers of sequences, integrating curated sequence alignments and phylogenetic trees, managing evolutionary analyses, and visualizing their output. *EGenBio *is organized into three conceptual divisions, *Evolution*, *Genomics*, and *Biodiversity*. The *Genomics *division includes tools for selecting pre-aligned sequences from different genes and species, and for modifying and filtering these alignments for further analysis. Species searches are handled through queries that can be modified based on a tree-based navigation system and saved. The *Biodiversity *division contains tools for analyzing individual sequences or sequence alignments, whereas the *Evolution *division contains tools involving phylogenetic trees. Alignments are annotated with analytical results and modification history using our *PRAED *format. A miscellaneous *Tools *section and *Help *framework are also available. *EGenBio *was developed around our comparative genomic research and a prototype database of mtDNA genomes. It utilizes MySQL-relational databases and dynamic page generation, and calls numerous custom programs.

**Conclusion:**

*EGenBio *was designed to serve as a platform for tools and resources to ease combined analysis in evolution, genomics, and biodiversity.

## Background

Large-scale genomic technologies have generated an extraordinary amount of data in the past few decades. Consequently, a huge effort has been made toward creating biological databases and systems to organize, analyze, and share information with the world-wide community [1-4]. The application of genomic technologies to molecular evolution has opened new frontiers in the interdisciplinary field of evolutionary genomics, and this has given rise to a great potential to elucidate complex questions in biology [2,5]. Understanding of evolutionary processes is critical, since they determine the sequence, structure, and function of macromolecules, and ultimately shape the higher-level biological complexity of organisms.

Genomic biodiversity has been defined as dense sampling of molecular data from diverse taxonomic groups for large genomic regions or complete genomes [6], and inferences concerning evolutionary processes are greatly improved by adopting combined molecular and computational approaches that include a large amount of genomic biodiversity [6-9]. We have found that the study of evolutionary genomics in the context of a dense sampling of species gives rise to many unique data processing problems, and so have developed the *E*volutionary *Gen*omics and *Bio*diversity (*EGenBio*) project as a web-based system to simplify large-scale evolutionary data management.

The central aim of *EGenBio *is to provide integrated analysis and visualization of raw sequence data, alignments, and phylogenetic trees, to rapidly curate and annotate that data, and to filter that data based on these annotations for further analysis of specific genomic contexts. It is designed to be robust to change and easily extensible to other datasets and other analytical programs. To accomplish this, *EGenBio *has web-based interfaces designed to: (1) access computational tools for phylogenetic and evolutionary analyses; (2) facilitate the construction of large-scale sequence and alignment datasets across diverse taxa; and (3) provide a framework for comparative analysis of diverse genes and genomes. *EGenBio *may also serve to promote the utility of increases in the scale of genomic biodiversity.

## Overview

**Figure 1:**
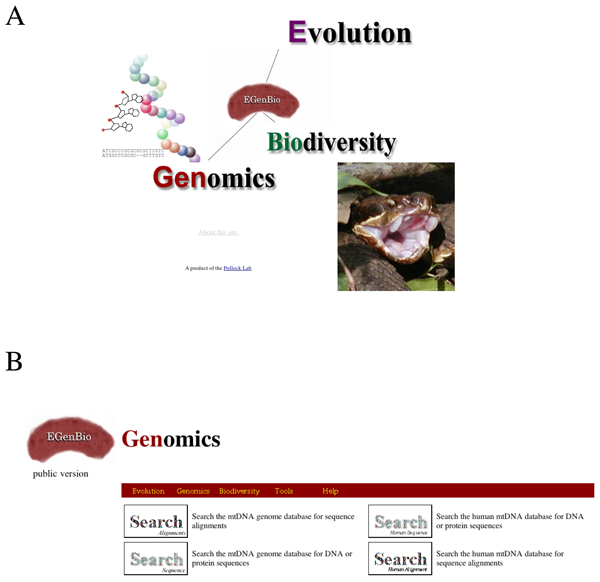
**Organization of *EGenBio***. *EGenBio *is accessed through a splash page that links to the three main divisions, *Evolution*, *Genomics*, and *Biodiversity *(A). Each division has its own access page to the tools that are organized in that division. For example, the *Genomics *division page is shown in (B). The division of each page is clearly marked to allow quick movement among divisions and to the *Tools *section and mirrored *Help *pages.

The three conceptual divisions of *EGenBio *(*Evolution*, *Genomics*, and *Biodiversity*) serve to organize web-based access to the primary custom-built tools (Figure 1, Table 1). The *Genomics *division provides access to pre-aligned sequence databases, and serves as a means of flexibly selecting genes and species/genomes of interest and producing a concatenated and annotated alignment for use in further analysis. The data is stored in a MySQL database and accessed "on the fly". Our prototype database contains complete vertebrate mitochondrial genomes that are mostly obtained from NCBI RefSeq annotations [10], but also contains "pre-submission" genomes provided by other investigators. Private access to "pre-submission" genomes allows users to pre-integrate their data with publicly available data for analysis in the primary genome publication, and is available on request. Complex searches can be stored, and registered users can create a personal list of searches that are retained in memory. Alignments for protein-coding genes are based on their amino acid sequences using ClustalW [11], but either amino acid or nucleotide alignments can be selected. The *Biodiversity *division is a collection of tools for analysis of alignments or sets of sequences (Table 1). These tools do not require a phylogenetic tree. Examples include lists of sequences available, database summaries, graphical representation of gene order information, and summary information on selected or uploaded alignments. This section also includes tools to aid experimental analysis of evolutionary genomics predictions, such as generating primers that represent all possible permutations of a set of sequences, or generating degenerate primers that reflect a sample from a posterior probability prediction of a particular ancestral sequence. The *Evolution *division includes tools (Table 1) that incorporate or extract information from phylogenetic trees and that translate from accession numbers to human-readable labels for sequences (e.g., genus species designations, or common names of organisms). This division also includes access to programs for processing and visualization of alignment filters (described below), coevolutionary analysis [12,13], and saturation mutagenesis analysis [14].

In addition to the three main sections, *EGenBio *contains a *Tools *section that serves as a repository for small stand-alone tools that may also exist as components of other pages, or which serve other simple purposes. The *Help *link leads not to a separate section, but rather to what we will call a separate "framework". The structure of the *Help *framework mirrors the main framework exactly, but instead of linking to actual tools, *Help *pages link to detailed descriptions and documentation for each page. Invisible to most users, a hidden *Design *framework allows for rapid editing and movement of page and site structure information from design to laboratory testing stages, and finally to public access.

## Discussion and Conclusion

*EGenBio *is a web-based system for analysis in evolutionary genomics and biodiversity. It provides tools and resources for quickly creating, modifying, and analyzing large alignment datasets in ways that we have found useful in our own computer-based and experimental evolutionary genomics research. Our prototype database of complete vertebrate mitochondrial genomes represents the densest complete set of genes currently available from closely related organisms. It can be accessed flexibly according to comma-separated queries or a phylogenetic tree navigation system. *EGenBio *is designed to be easily extensible to use with other protein complexes and other analytical programs. Our goal is to incorporate and utilize as many existing programs as possible, and to develop only "added value" programs. In the current public version, all tools are novel to our system except that alignments are created using ClustalW [11]. The *PRAED *alignment annotation system based on data filters allows alignments to be modified easily according to user interest in annotation features, and allows for the results of analyses to be returned as further annotations on the alignments. Since it is derived from the *NEXUS *format, it is easy to add batch commands to direct analyses using many common phylogenetic analysis programs. The *PRAED *format and data filters are a unique feature of the *EGenBio *system.

Future modules under development in *EGenBio *include the creation of additional data filters, incorporation of more genes for analysis of functional divergence, development of further visualization tools for statistical analyses of evolutionary dynamics, and automated procedures for analysis using existing programs and tools. We also welcome feedback from the scientific community on areas of general need for integrated evolutionary genomics tools. *EGenBio *is publicly available and can be accessed at  via anonymous login. User accounts that allow users to save search parameters and results are provided upon request. Incorporation and private access to pre-publication data can also be accommodated upon request. Replication of the *EGenBio *system would require a Linux-based operating system capable of running Perl, Perl-GD, R, PHP, MySQL, and an Apache web server. It would also require installation of numerous custom scripts in addition to ClustalW.

## Abbreviations

LRT: Likelihood ratio test; MCMC: Markov chain Monte Carlo; mtDNA: mitochondrial DNA; NCBI: National Center for Biotechnology Information; *PRAED*: *PR*agmatic *A*nalysis of *E*volutionary *D*ata.

## Authors' contributions

LAN is a scientific database curator responsible for the design and documentation of the *Help *and *Design *pages and organism database, and co-wrote this manuscript. MTR developed and managed the system and has created numerous tools. ZOW developed one of the tools and assisted in preparation of the manuscript. JJF began initial development of the system and developed several tools. RJ worked on the *Help *and *Design *pages, the mirror/framework system, and automated generation of pages. ZJJ developed one of the tools and assisted in preparation of the manuscript. TJM assisted in preparation of the manuscript, and review and editing of the web site, and is developing one of the tools. DDP is the principal investigator responsible for the creation, conceptualization, and management of the *EGenBio *system, developed early versions of many of the tools, and co-wrote this manuscript.

## Figures and Tables

**Table 1:** Custom tools* currently in the main divisions of *EGenBio*

**Division**	**Tool Name**	**Tool Description**
*Genomics*	*SearchSequence*	Search the mtDNA genome database for DNA or protein sequences
*Genomics*	*SearchAlignment*	Search the mtDNA genome database for sequence alignments
*Genomics*	*SearchHuSequence*	Search the human mtDNA database for DNA or protein sequences
*Genomics*	*SearchHuAlignment*	Search the human mtDNA database for sequence alignments

*Evolution*	*TranslateTree*	Translate labels of a tree file
*Evolution*	*FilterViz*	Visualize filters associated with alignments
*Evolution*	*TreeReader*	Extract tree clusters along with information on branch lengths
*Evolution*	*SaturationTool*	Visualize results from saturation mutagenesis MCMC analysis
*Evolution*	*LnLCorr*	Detect coevolution among residues using LRTs and trees

*Biodiversity*	*SpeciesList*	List species currently in *EGenBio*
*Biodiversity*	*SpeciesSearch*	Search species by taxonomic group or NCBI genome identifier
*Biodiversity*	*LocusOrder*	Display mitochondrial gene order for specified taxa
*Biodiversity*	*DatabaseSummary*	Provide information about the *EGenBio *databases
*Biodiversity*	*PrimerPermuter*	Generate permutations for use in primer design
*Biodiversity*	*PrimerAlternatives*	Produce degenerate primers that reflect amino acid variation

*All tools listed are original to *EGenBio*, except that alignments are based on ClustalW [11].
